# Activity Learning as a Foundation for Security Monitoring in Smart Homes

**DOI:** 10.3390/s17040737

**Published:** 2017-03-31

**Authors:** Jessamyn Dahmen, Brian L. Thomas, Diane J. Cook, Xiaobo Wang

**Affiliations:** 1 School of Electrical Engineering and Computer Science, Washington State University, Pullman, WA 99164, USA; jb3dahmen@wsu.edu (J.D.); bthomas@eecs.wsu.edu (B.L.T.); 2 FutureWei Technologies, Inc., Santa Clara, CA 95050, USA; xiaobo.sc.wang@huawei.com

**Keywords:** security monitoring, activity learning, anomaly detection, smart home automation

## Abstract

Smart environment technology has matured to the point where it is regularly used in everyday homes as well as research labs. With this maturation of the technology, we can consider using smart homes as a practical mechanism for improving home security. In this paper, we introduce an activity-aware approach to security monitoring and threat detection in smart homes. We describe our approach using the CASAS smart home framework and activity learning algorithms. By monitoring for activity-based anomalies we can detect possible threats and take appropriate action. We evaluate our proposed method using data collected in CASAS smart homes and demonstrate the partnership between activity-aware smart homes and biometric devices in the context of the CASAS on-campus smart apartment testbed.

## 1. Introduction

Smart homes have long held the promise of making our everyday environments secure and productive. Individuals spend the majority of their time in their home or workplace [1] and many feel that these places are their sanctuaries. In order to preserve that feeling, smart homes can make use of technologies such as embedded sensors and machine learning techniques to detect, identify, and respond to potential threats. While stand-alone security systems have been used in homes for many years, they cannot make adequate use of the rich information that is available from sensors integrated throughout the home and algorithms that can reason about normal and abnormal behavior in the home.

In this paper, we introduce a smart-home based approach to home security. Our proposed approach is built on the foundation of the CASAS smart home system, in which sensors are embedded in the environment. The sensors collect information about the state of the home and resident. Activity learning techniques use this information to identify and reason about routine or normal behavior in terms of recognized and forecasted activities. This identified behavior forms the basis for threat detection based on sensing abnormal behavior. Once the abnormal behavior is identified as a threat, the home selects an action to take as a response.

We evaluate our approach in the context of actual smart home testbeds. The CASAS smart home infrastructure is described in the next section and has been deployed in over 100 residences. We use actual and synthetically-manipulated data from three single-resident smart homes (named B1, B2, and B3) to evaluate our approach to threat detection and response. In addition, we demonstrate the automated-response smart home using data from a multi-resident on-campus smart apartment testbed (named Kyoto). Finally, we describe challenges and strategies for enhancing and utilize smart home-based security technologies.

## 2. CASAS Smart Home

**Figure 1:**
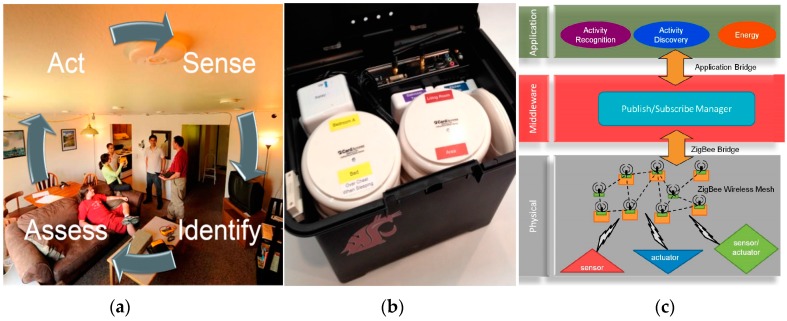
(**a**) CASAS smart apartment testbed and project goals, (**b**) SHiB smart home in a box, (**c**) smart home software infrastructure.

Our proposed secure smart home is built on the foundation of the smart home infrastructure developed at the Center for Advanced Studies in Adaptive Systems (CASAS). The smart home sense, identify, assess, and act functions represent a continuous cycle (see Figure 1). Using embedded sensors, the home senses the state of the physical environment and its residents. Software provides reasoning capabilities to identify behavior and assess the well-being of residents (Figure 1a). Finally, the home can act on its information in order to ensure the safety, security, comfort, and productivity of the residents. The CASAS infrastructure (Figure 1c) includes components at the physical layer to sense and act on the environment. The components at the middleware layer provide component communication, identification, and synchronization. Finally, the components at the application layer provide specialized services such as activity learning, sensor fusion, and optimization for specific smart home goals. The smart home components communicate with each other via software “bridges”, or communication links. Examples of such bridges are the Zigbee bridge to provide network communication, the Scribe bridge to store messages and sensor data in a relational database, and bridges for each application.

**Figure 2:**
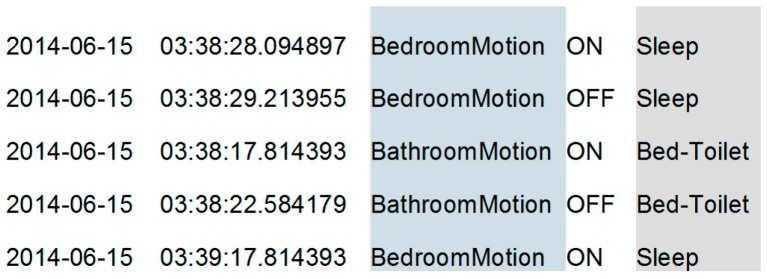
Raw sensor data includes a timestamp, sensor identifier, sensor message, and an automatically-generated activity label.

The smart home testbeds we use to evaluate our proposed secure smart home rely on the streamlined CASAS “smart home in a box”, or SHiB (Figure 1 middle). The locations of each SHiB sensor are predefined in terms of functional areas of the home, which supports the creation of generalizable activity models. Each CASAS SHiB is installed by our team or the residents themselves [2], generally in less than two hours, and can be removed in less than twenty minutes. The lightweight nature of the design allows us to evaluate new software methods, such as the ones described in this paper, outside of laboratory or simulation settings, which is a challenge for many smart home and activity learning projects [3,4]. While each smart home site runs independently, the homes can also securely upload events to be stored in a relational database in the cloud. Figure 2 shows a sample of collected smart home data with associated automatically-generated activity labels.

**Figure 3:**
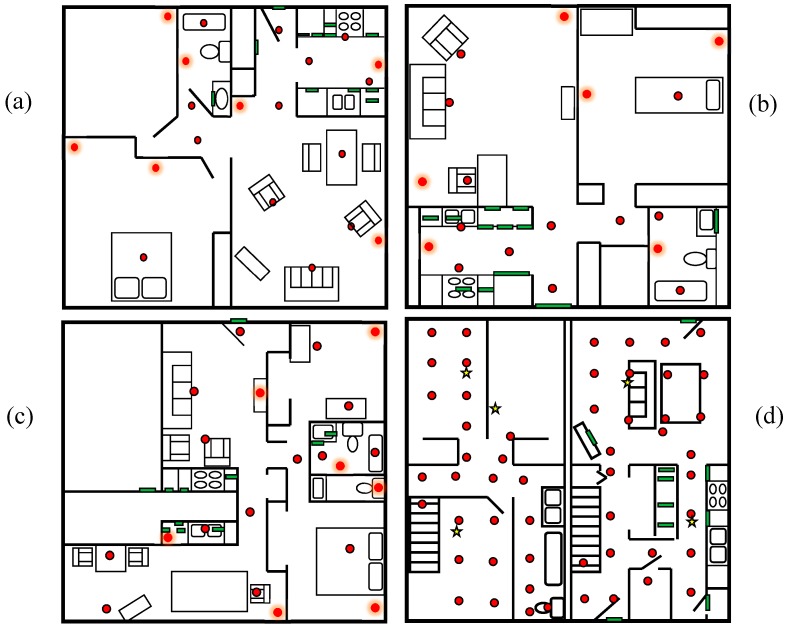
(**a**) B1; (**b**) B2; (**c**) B3; and (**d**) Kyoto smart home testbeds with floorplan and sensor locations. Red dots indicate motion sensors, green rectangles indicate door/temperature sensors, and yellow stars indicate ambient temperature sensors.

We evaluate our proposed secure smart home using four of the CASAS testbeds. The sensor layouts for these testbeds are shown in Figure 3. As shown in the figures, data is collected by sensors that monitor infrared motion (red circles), door open/close status (green rectangles), and ambient temperature as well as and ambient light (yellow stars). The B1, B2, and B3 testbeds each housed one resident and the Kyoto testbed housed two residents. We modeled the activities Bathe, Bed-toilet transition, Cook, Eat, Enter home, Leave home, Personal hygiene, Relax, Sleep, Take medicine, and Other activity.

## 3. Activity Learning

Our approach to threat detection in smart homes is unique because it incorporates knowledge of current and activities to determine deviations from normal behavioral patterns. Those deviations represent potential threats that require further investigation and response. Most cyber-physical systems generally use a fixed set of parameters such as time and location to identify a current context, and this context forms the basis for many context-aware services including security services. Not only is this parameter set fairly small, but the parameters are typically considered separately. We postulate that learning activities provides a richer source of information for smart homes and can thus improve the security of the home. Furthermore, we maintain that an activity-aware home will provide even more powerful security services than one that is simply aware of resident locations and movements, because it can detect deviations from learned complex activity patterns.

Learning and understanding observed activities is essential for enabling secure smart homes to be sensitive to the needs of the humans that inhabit them. Our smart home makes use of activity learning in order to transform the system into one that is activity aware. Here, we describe the activity recognition and discovery algorithms that create the foundation of activity awareness in the secure smart home.

### 3.1. Activity Recognition

Activity recognition algorithms label activities based on the data that are collected from sensors in the environment. Once this information is provided, we can identify situations that are relevant to home security such as sleeping, entering/leaving the home, cooking, and performing activities that use valuable items. The goal of an activity recognition algorithm is to map a sequence of sensor readings, or sensor events, *x =* <*e_1_ e_2_ ... e_n_*>, onto a value from a set of predefined activity labels, *a* ∈ *A*. Activity recognition can be viewed as a type of supervised machine learning problem. We further assume the availability of a feature function, *Φ*, that can compute a d-dimensional feature vector from a sequence of sensor events. Our activity recognition algorithm, CASAS-AR, learns a function *h* that maps a feature vector, *X* ∈ *R^d^*, describing a particular sensor event sequence onto an activity label, *h:X*→*A*. CASAS-AR can use the learned function to recognize and label occurrences of the learned activity.

**Figure 4:**
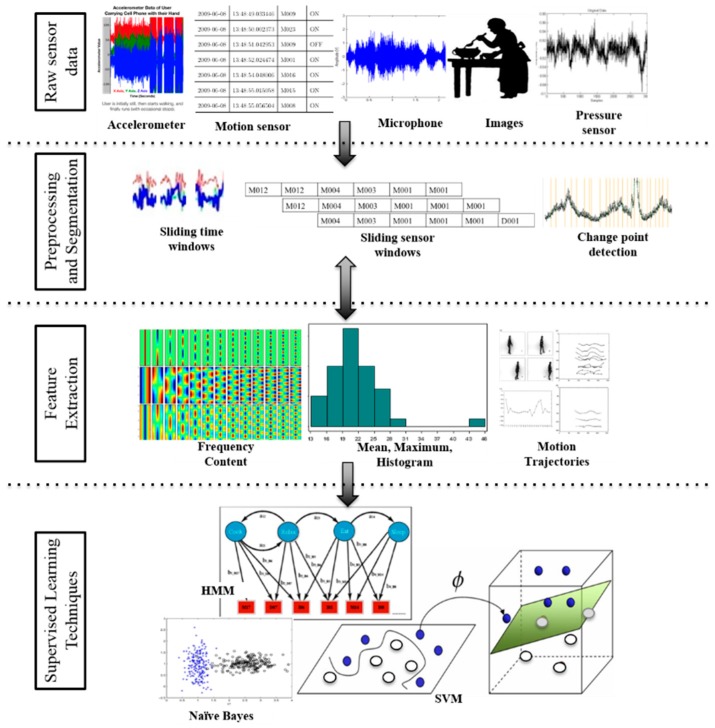
Activity recognition includes stages of raw sensor data collection, data preprocessing and segmentation, feature extraction, and supervised machine learning.

There are challenges in activity recognition that are unique among machine learning problems. The input data is often sequential and noisy, the data is not clearly partitioned into activity segments, and the data is occasionally multi-label. Some of these challenges are addressed through additional data processing such as the steps shown in Figure 4 which include collecting and preprocessing sensor data, dividing it into subsequences of manageable size, then extracting subsequence features. The final feature vectors are either labeled by an expert to use as training data or are input to an already-trained model to generate the corresponding activity label. The raw data we collect in smart homes together with the features we use to learn activity models from smart home data are summarized in Table 1. In contrast to sampling-based sensors that generate a continuous stream of values, all of the smart home sensors detect discrete events. As such, they generate text messages with sensor values, or events, only when the sensor internally notes a change in state.

The activity recognition approach that we incorporate into our secure smart home builds upon our prior work to design algorithms that automatically build activity models from sensor data using machine learning techniques [5,6,7,8]. Other groups have also explored a large number of approaches to perform supervised activity recognition [9,10,11,12,13,14,15,16,17,18,19,20,21,22,23]. These have been tested for a variety of sensor modalities, including environment [7,24,25], wearable [26,27,28], object [29,30], smart phones [31,32], and video [33]. The learning methods can be broadly categorized into template, generative, discriminative, and ensemble approaches. Template matching techniques employ a pattern-based classifier such as k-nearest neighbors, often enhanced with dynamic time warping to a varying window size [34]. Generative approaches such as naïve Bayes classifiers, Markov models and dynamic Bayes networks have yielded promising results for behavior modeling and offline activity recognition when a large amount of labeled data is available [7,35,36,37,38,39,40,41]. On the other hand, discriminative approaches that model the boundary between different activity classes offer an effective alternative. These techniques include decision trees, meta classifiers based on boosting and bagging, support vector machines, and discriminative probabilistic graphical models such as conditional random fields [7,42,43,44]. Other approaches combine these underlying learning algorithms, including boosting and other ensemble methods [36,45,46].

While many activity recognition algorithms have been proposed, they are typically designed for constrained situations with pre-segmented data, a single user, and no activity interruptions. Our recent work has extended this to consider generalization of activity models over multiple users. We use this generalized model in this paper to provide activity labels for the collected sensor data in each of the smart homes. To facilitate learning such a general model, we utilize a common vocabulary of sensor types and locations that allows us to design algorithms that recognize activities even in new spaces with no training data. Because we label data in real time, we do not employ the offline data segmentation found in other approaches [29,47,48,49,50,51,52,53]. Instead, we move a sliding window through the sensor data in real time, extracting features from the window of data and mapping them to an activity label that represents the current activity (or activity corresponding to the last reading in the window). Utilizing a random forest of decision trees, we have achieved >95% recognition accuracy for as many as 33 activities in over 20 homes [38,54] including those we include in the evaluation of our proposed secure smart home.

### 3.2. Activity Discovery

In order to offer an activity-aware smart home, we need to enhance activity recognition research in a novel way by combining activity recognition and discovery. The most common approach to reasoning about human activities from sensor data is to identify activities that are interesting to track, then model those activities. However, to model and recognize activities, a large amount of sensor data must be available that is pre-labeled with ground truth labels. For most activities that are performed in real-world spaces, such pre-labeled data is not readily available. As a result, the set of commonly-tracked activities represents a fraction of an individual’s total behavioral routine [55,56]. Tracking such a small number of activities can affect learning performance because the remainder of the data provides important context and putting unlabeled activities into an “Other activity” category yields a significant class imbalance. Considering the datasets we evaluate in this paper, the “Other activity” category represents more than half of the total sensor data. This situation is particularly problematic because the “Other activity” category represents a potentially boundless combination of diverse activities and is therefore difficult to model accurately.

While activity discovery can be used in many varied ways for smart home security, here we use it specifically to address the class imbalance problem. Specifically, we introduce intra-class clustering as a preprocessing step for activity recognition. Here, majority classes (such as “Other activity”) are decomposed into sub-classes. Thus, activity class ai ∈ A is decomposed into sub-classes Sai = {ci1, ..., cik}. Training instances are assigned new class labels corresponding to their respective sub-classes and used to build the new activity models. The classifier will predict activity labels using both predefined and discovered sub-class labels. When the learned models are used to label new data, the sub-class labels are mapped back onto the original parent class labels (in this case, “Other activity”). Multiple types of clustering algorithms can perform this function. Some require a predefined number of clusters such as k-means++ [57], in which case we choose the number of clusters so that the resulting subclasses are close to the mean activity size for balance, while others such as CascadeSimpleK-Means [58] partition data based on natural groupings. Our previous research [59] has indicated that intra-class cluster frequently outperforms sampling-based methods for imbalanced class distributions. In our secure smart home, we utilize k-means++ to perform intra-cluster clustering in order to enhance the CASAS-AR activity recognition.

## 4. Anomaly Detection

One approach to designing secure smart homes is to train the home to recognize specific situations such as resident falls. This approach can be effective if enough realistic labeled data is available to emulate each possible situation. However, gathering this type of data in actual homes is difficult and can be a limitation for this approach. Furthermore, looking at predefined target situations prevents the home from detecting a broader range of security threats.

In order to find a larger collection of possible threats, we turn our attention to anomaly detection. As Chandola states, anomaly detection is “the problem of finding patterns in data that do not conform to expected behavior” [60]. One of the important roles a smart home can play is to automatically find anomalies. Anomalies can be viewed as a threat to an individual’s safety in the home or even as a threat to their health [61,62,63,64,65].

There are several standard techniques for finding anomalies or outliers that are commonly employed. One such technique is to cluster data points into groups based on their distance to the cluster center. Once the data is clustered, points that are far from all of the cluster centers can be labeled as outliers and can be considered as anomalies. In cases where the data is normally distributed, z scores can also be computed. The z score for any data point is its distance from the sample mean, divided by the sample standard deviation. All z scores greater than 3.5 are typically considered to be outliers. The problem with employing clustering methods is that the clustering algorithms rely upon calculating distances between data points. While this can be effective for univariate data, smart home sensors generate multi-variable, complex data and Euclidean distance-based methods do not always reflect the important aspects of the collected data.

To address this situation and detect anomalies in smart home sensor data, we employ an isolation forest [66] to generate anomaly scores, where a higher score indicates a greater deviation from normal data patterns. An isolation forest builds a decision tree to recognize data points that fit into well-known classes. Because decision tree algorithms attempt to build the smallest possible decision tree that is consistent with training data, an outlier can be observed as a data point x that has an unusually long path length from the root of the decision tree to the leaf node containing the data point, or h(x). Each branch along the paths queries the value of a particular feature, thus this approach to anomaly detection utilizes the same feature vector employed by activity recognition and is sensitive to activities occurring in the home.

**Figure 5:**
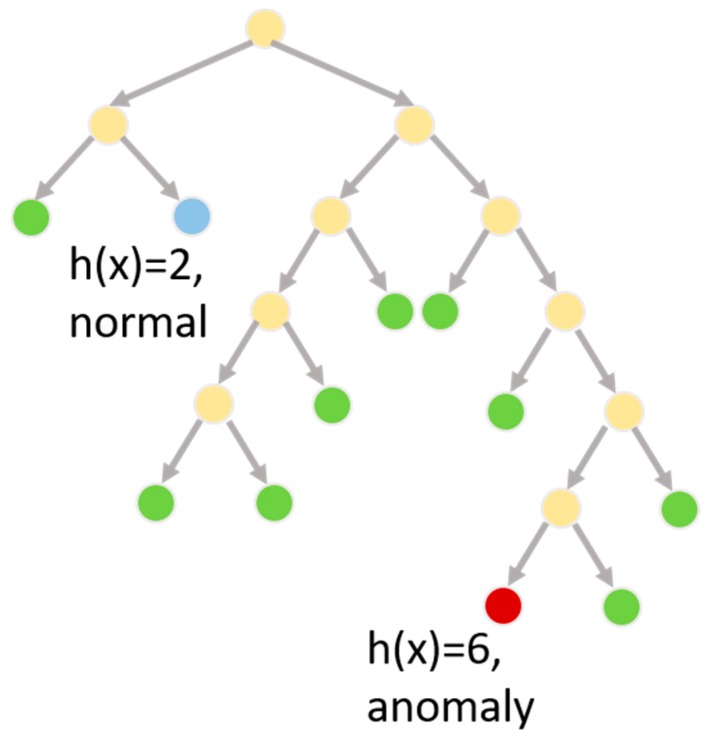
Path lengths are averaged over a collection of decision trees to determine the anomaly score for a smart home sensor data sequence.

To generate an anomaly score, a set of decision trees is trained on the same data, where a node’s children for each tree in the set are created by randomly selecting a feature to query and randomly selecting a split point for the feature values. Multiple trees are created, reducing the tendency to overfit and increasing the likelihood that a point with a large average *h*(*x*) value, or *E*(*h*(*x*), is a true anomaly (see Figure 5 for an example decision tree and *h*(*x*) value). The average height is normalized across trees dividing the value by *c*(*n*) = 2*H*(*n* − 1) − (2(*n* − 2)/*n*), where *H*(*i*) is the harmonic number, n represents the number of leaf nodes in the tree, and the function *c*(*n*) reflects the average path length of an unsuccessful search in a binary search tree. The anomaly score s of a data point x is thus defined as shown in Equation (1).

(1) $$s(x,n)=2^{-\frac{E(h(x))}{c(n)}}$$

This method has been effective at detecting anomalies for many varied applications, even on streaming data [67]. We report anomalies as the data points with the top anomaly scores. Selecting a threshold of scores to report as anomalies is application dependent. If the threshold is too high, some anomalies may be missed, thus reducing the number of true positive anomalies that are detected. If the threshold is too low then many normal situations will be reported as anomalies, increase the number of false positives. In an empirical testing of our approach, we experimented with different fractions of outliers to report and found that reporting 0.1% yields successful detection of most known anomalies without generating an excess number of false positives.

## 5. Evaluation

Our proposed secure smart home technology is built on the notion of activity-aware anomaly detection. In this section, we analyze the effectiveness of this proposed approach which is applied to detecting anomalies and security threat conditions. To do this, we utilize actual smart home data collected in home testbeds described in Section 2. We first validate the ability of our algorithm to discover known anomalies then evaluate the technology for security-related threat conditions in an on-campus smart home testbed.

### 5.1. Hybrid Real-Synthetic Data Generator

We evaluate our smart home security detection system on data collected from real smart homes. This allows us to determine the usefulness of the method on actual smart home data as residents perform their normal daily routines. In real-world daily situations, however, anomalies are rare and are not always well documented. Therefore, we not only test the data on untouched real-world data but we also find a need to create synthetic data that is reflective of real-world data and then modify the synthetic data to contain known anomalies.

To create hybrid real-synthetic data, we designed a Markov model-based generator. The data generator is designed to be probabilistically similar to the original smart home datasets, yet allow some variation that could be detected as anomalies depending upon the methods that are used. A Markov model is a statistical model of a dynamic system. The system is modeled using a finite set of states, each of which is associated with a multidimensional probability distribution over a set of parameters. Transitions between states are also governed by transition probabilities. The parameters for our model are the possible sensor event states in the home. We utilize a hidden Markov model (HMM), so the underlying model is a stochastic process that is not observable (i.e., hidden) and is assumed to be a Markov process (i.e., the current state depends on a finite history of previous states). The Markov property specifies that the probability of a state depends only on the probability of the previous state. More “memory” can be stored in the states, however, by using a higher-order Markov model. In our case, we employ a third-order Markov model, which means that the probability of the next state *xi* + 1 depends on the previous three states, as shown in Equation (2).

*P*(*x*_*i*+*i*_|*x_i_, x*_*i*−1_*, x*_*i*−2_*, .., x*_1_) = *P*(*x*_*i*+1_|*x_i_, x*_*i*−1_*, x*_*i*−2_) (2)

To generate synthetic data, we learn the structure of our HMM from the real smart home data [55] then use the learned structure to synthesize data that is reflective of the original data but may contain minor variations. As a result, we can generate an arbitrary amount of data and expect that few if any anomalies would be found that do not exist in the original real-world datasets. HMMs are well-suited to this type of data generation and have been used for this task in applications including speech synthesis, gene sequences, and cognitive processes [56,57,58].

### 5.2. Validation of Anomaly Detection

Our first evaluation step is to validate the accuracy of our anomaly-based smart home security threat detection approach. The second step is to demonstrate its use in a security-based smart home scenario. To validate our activity anomaly-based approach, we generate one week of hybrid real-synthetic data for the B1, B2, and B3 smart homes shown in Figure 3. We use one day of real data for each site to generate a week’s worth of hybrid synthetic data. This allows us to keep the baseline data fairly uniform while allowing for some minor variation in residents’ activities, locations, and timings throughout the dataset. In each of these datasets, we pick 20 random spots where the generated data is modified to simulate an anomaly. Each anomaly spans 30 events (the size of our activity recognition sliding window) and is created by randomly modifying the data while maintaining the same dates and times as the original data.

In order to determine the role that activity awareness plays in anomaly detection, we perform anomaly detection with alternative feature vectors. The tested feature vectors are listed below.

- f.standard: include timing, window, and sensor features that are listed in Table 1
- f.activity: include timing information and the activity label for the corresponding window of sensor data as provided by the discovery-enhanced CASAS-AR activity recognition algorithm
- f.all: include all of the standard features together with activity label

To measure performance, we compute true positive rates and false positive rates for the algorithm based on its ability to detect the known anomalies. We compute these rates as we vary the fraction of outliers that are reported as anomalies, from 0.1% to 0.5%. We can then use the rates computed for each of these thresholds to generate a receiver operating characteristic (ROC) curve and report the Area Under the ROC curve, or AUC. This allows us to not only determine how well the algorithm is performing overall but to compare the alternative feature vectors and thus assess the impact of activity awareness on our proposed anomaly detection method.

The results from this experiment are summarized in Table 2. As the true positive rates show, most of the embedded anomalies are found for each dataset. The B1 dataset presents the easiest case for detecting anomalies because the resident had a very structured daily routine with predictable activities, times, and locations. Even with this amount of regularity, however, the anomaly detector does find quite a few anomalies in the normal data. To an extent, this is to be expected, because humans are unpredictable and their routines are highly variable. This presents a challenge for anomaly detection in general because an algorithm that is robust enough to find true anomalies in the data will also detect such natural variations. The B3 dataset was the most challenging for finding the embedded anomalies. This is due, in part, to the fact that the normal data itself contained some anomalous situations, with one of the door sensors reporting a very large number of repeated OPEN/CLOSE messages over short periods of time. In the face of these preexisting anomalies, the embedded anomalies were not always as obvious.

For all of the datasets, we observe that activity awareness improves the ability to detect anomalies. In the case of the B1 and B2 datasets, the improvement in AUC values when using standard features and activity labels is significantly better (*p* < 0.01) than using standard features alone. In the case of the B2 dataset, the activity labels alone were particularly effective at discovering almost all of the embedded anomalies.

### 5.3. Security Application

We begin the second half of our evaluation with a description of a security case study scenario. A secure smart home is valuable for monitoring the state of the home when the resident has left. An activity-aware smart home can recognize when the resident has left and when a person enters the home. For this type of security monitoring, a camera can also be used to perform face recognition on individuals who enter the residence. However, many residents do not want a camera monitoring them continuously. Additionally, face recognition techniques encounter difficulties when the person is obstructed or there are poor lighting conditions. In our scenario, the smart home sensors monitor the behavior of a person when they first enter the home. When the behavior is significantly different from normal in this context (is anomalous), then the home can automatically respond by turning on the camera and face recognition software.

In this section, we demonstrate the ability of our secure smart home to perform these tasks. We evaluate the ability of the anomaly detection algorithm to detect threats that fit this scenario. We then illustrate how our smart home testbed performs for our case study scenarios. For this evaluation, we utilize the Kyoto smart home testbed shown in Figure 3. We have collected data in this testbed for almost ten years and have labeled the data with activities using the CASAS-AR software. We extracted 762 instances of the “Enter home” activity and five minutes of sensor data following recognition of this activity. We filtered instances when the home was vacant or used extensively for other smart home studies. Each of these instances represents a single data point. We then generate a feature vector using the features summarized in Table 1 except for window duration, which is fixed in this case. Because the residents did not report any security issues during the data collection, we treat these data points as normal.

Next, we insert three sample scenarios into the data by generating sensor data that fits dates and times for actual “Enter home” activities but which represents three known conditions, one which is normal and two which are anomalous. In the first scenario, an individual enters the home through the front door (Figure 2 middle bottom of Kyoto floor plan), goes into the kitchen (Kyoto bottom right), and puts away groceries. The scenario triggers door sensors for the front door as well as the kitchen pantry and cabinets, and triggers motion sensors in the entryway and kitchen. In the second scenario, an individual enters the front door and quickly moves through the entire lower floor of the apartment, opening all of the closet and cabinet doors as though search for items to steal. This scenario should be detected as an anomaly and a possible security threat (and thus cause the camera to turn on and other appropriate actions to be taken). In the third scenario, an individual enters the apartment by a means other than the front door. For our smart home, this is represented by the individual entering the apartment through the back door. Because the front door is closest to the parking lot, the back door is not typically used to enter the apartment. Entering the apartment through a window, or for this home the back door, is anomalous and again would be viewed as a security threat.

As in the previous section, we run our anomaly detection algorithm on the actual smart home data and three inserted scenarios. We utilize a threshold of 0.1% which we selected empirically based on its consistent performance for smart home data. There are 765 data points, two of which are known anomalies. Both anomalies are detected by the software.

The complete set of results is summarized in Table 3. As the table shows, the anomaly detection algorithm was fairly conservative and only detected seven anomalies. All of the data points that were not detected as anomalies fell correctly into the baseline (normal activity) category, including the scenario case that represented normal putting-away-groceries behavior. In five of the cases, however, baseline data was labeled as anomalous. We analyzed each of these data points to determine which it was selected as an anomaly and possible security threat. In three of the cases, the individual opened and shut many of the downstairs closet doors, creating an activity profile that was very similar to our second scenario and that was unusual for the residents. The date for these data points was at the end of the spring semester when the resident was packing to move out of the apartment. These are indeed anomalous situations although may not be considered a security threat. In the other two cases, the data again included many motion sensors throughout the residence. Because an area of the home was accessed that contained much of the smart home data collection equipment, these may have represented times in which the CASAS team was performing maintenance or upgrades.

**Figure 6:**
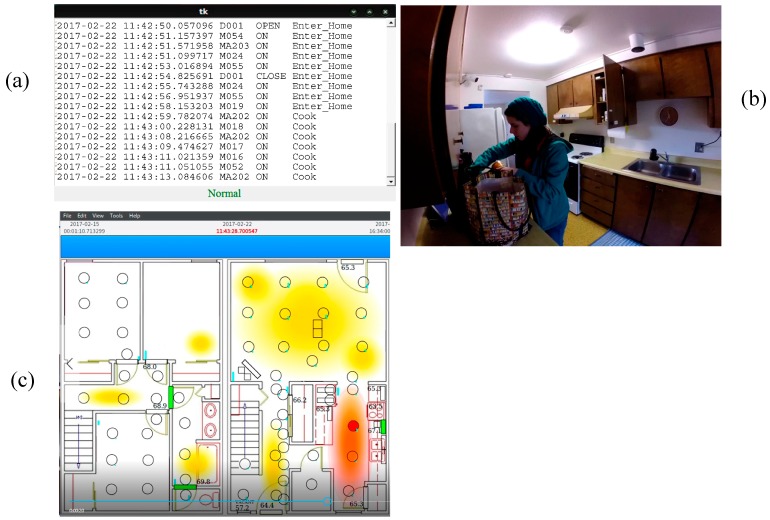
Screen shots from scenario 1 in the secure smart home. (**a**) Each sensor event is displayed on the computer screen. (**b**) The status bar at the bottom of the window indicates that this is normal behavior as the resident enters the home and puts away groceries. (**c**) Our real-time sensor event visualizer shows the status of sensors throughout the home during the scenario. This visualizer shows that the resident is in the kitchen (lower right area of the home) and the cabinet is open while she puts away groceries.

**Figure 7:**
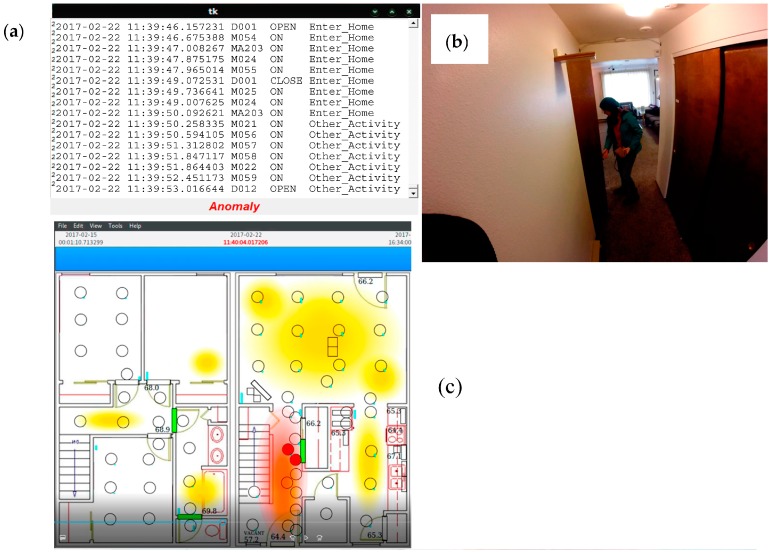

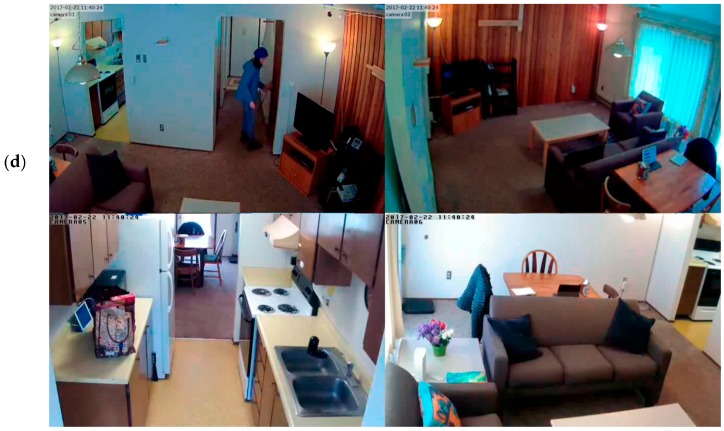
Screen shots from scenario 1 in the secure smart home. (**a**–**c**) A person enters the home and begins looking through closets and cabinets, which are sensed by the smart home (**d**) The home detects this as an anomalous situation and brings up the cameras in the home, which show the identity of the person and more details of what they are doing (in this case, removing a purse from the closet).

Finally, Figure 6 and Figure 7 show screenshots of our actual secure smart home system. We placed cameras throughout the home and captured video as a volunteer performed our three case study scenarios in the smart apartment. The screens indicate when the home is empty, when an individual enters the home, and whether the enter-home activity is normal or anomalous. In the two detected anomalous situations, the cameras are activated and a window pops up on the screen that shows the resident the captured video. At this point, face recognition software can be employed or the resident can view the video manually to determine whether the situation is a security threat that requires further action. We also include images from PyViz [59], our tool that shows the status of sensors in the smart home in real time as the resident moves throughout the space.

## 6. Related Approaches to Secure Smart Homes

As is indicated in Figure 1, secure smart homes need to sense the state of the environment, identify and assess possible threats, and act on those threats. While the approach we have described in this paper is unique in investigating an activity-predictive approach to secure smart homes, the work is one of many research and commercial efforts that have been developed to offer personal and home security in everyday residences [68].

In terms of sensing the state of a home, there are a number of technologies that provide sensor data specifically for security purposes. The most common sensing mechanism for home security is video cameras, and companies including iControl, Nest, SmartThings, Vivint, and Ring have enhanced traditional camera systems to offer home security features. These features include alerting homeowners of detected activity and connecting the camera with the home’s doorbell or other sensors [69,70]. Also common is the use of audio, which has been employed by Zhuang et al. [71] to detect falls and Moncrieff et al. [72] to detect unusual home noises. Biometrics have also been integrated into buildings to recognize individuals based on unique anatomical traits including voice, gait, retina, and facial features [73,74], as well as body shape (anthropometry) [75], footstep shape [76], body weight [77], and heart beat pattern [78].

These sensing mechanisms offer rich, fine-grained data, although for a limited view range. The depth of information also makes them not only a useful method of detecting threats but also a possible risk for invasion of privacy. In addition, the technologies rely on residents to interpret the collected data and to select an appropriate action in response to the situation.

In the area of identifying and assessing threats, many researchers take an approach similar to the one we propose by looking for outliers in sensor data patterns and viewing such outliers as threats to well-being. Unlike our approach, these previous methods do not look at activities and the related patterns. However, existing approaches have looked for outliers based on overall movement in the home [79], resident locations in a home [58,80,81], sensor event times [82], or sensor values [83].

As an alternative approach, some investigators predefine the types of threats that are of interest, then train systems to recognize those threats. For example, Teoh and Tan [84] designed a machine learning method specifically to recognize intruders based on input from a large variety of sensors including motion sensors, closed-circuit televisions, radio frequency identification (RFID) tags, magnetic contact switches, and glass breakage sensors. Aicha et al. [85] also look for these situations by analyzing transition times from when a home is empty to when an individual enters the home. A primary challenge for these types of systems that use supervised learning to detect highly specialized situations is that sufficient training data is normally not available for realistic home situations. For health applications, Han et al. [86] look for changes in the amount of time spent moving around the house, eating, sleeping, and performing hygiene. Williams and Cook [87] also look for changes in waketime and sleeptime behavior as a means to detect and circumvent sleep disturbances. In other approaches, researchers have constructed methods to look for changes in activity times for a constrained set of rule-based activity patterns [88,89,90,91].

## 7. Conclusions

In this paper, we introduce an activity-aware approach to creating secure smart homes. Smart homes have become adept at robustly and unobtrusively collecting sensor data that provides insights on human behavior. When this data is labeled using activity recognition algorithms, the smart home can become activity aware. We demonstrate how this information can be employed by an anomaly detection algorithm to detect security threats in real time. We validate our approach by detecting known anomalies in hybrid real-synthetic smart home data and demonstrate its use in a security home case study.

While this work demonstrates that smart homes can be automated for security threat detection, there are still many avenues for ongoing research. One problem that we highlighted in our experiments is that many anomalies naturally exist in human behavior data and not all of these represent security threats. In our future work, we will involve smart home residents to train the smart home on the types of anomalies that are of interest. We will also design an ensemble approach that employs multiple types of anomaly detection, including detection of location-based anomalies, current activity anomalies, and forecasted activity anomalies to improve the performance of anomaly detection. Finally, we will incorporate automated face recognition and resident notification to automate not only detection of anomalies but response to security threats.

## Figures and Tables

**Table 1:** Raw smart phone data, features, and activity class descriptors.

Domain	Number	Types of Features
*Raw Sensor data*	4 types	infrared motion (ON/OFF); magnetic door (OPEN/CLOSE); ambient light (continuous); ambient temperature (continuous)
*Timing features*	3 features	day of week; hour of day; seconds past midnight
*Window features*	9 features	most recent sensor in window; first sensor in window; window duration; most frequent sensors from previous two windows; last sensor location in window; last motion sensor location in window; entropy-based data complexity of window; time elapsed since last sensor event in window
*Sensor features (n sensors in home)*	2n features	count of events for each sensor in window; elapsed time for each sensor since last event

**Table 2:** Average true positive rate (TPR), average false positive rate (FPR), and Area Under Curve (AUC) values for the B1, B2, and B3 datasets with alternative feature vectors.

Dataset	Features	Average TPR	Average FPR	AUC
***B1***	*f.standard*	1.0000	0.0008	0.73
	*f.activity*	1.0000	0.0021	0.68
	*f.all*	1.0000	0.0008	0.87
***B2***	*f.standard*	0.8000	0.0021	0.49
	*f.activity*	0.5000	0.0032	0.42
	*f.all*	1.0000	0.0011	0.79
***B3***	*f.standard*	0.4500	0.0044	0.47
	*f.activity*	0.7000	0.0040	0.40
	*f.all*	0.8500	0.0038	0.47

**Table 3:** Performance of activity-aware anomaly detection in smart home security scenarios.

True Positives	False Positives	True Negatives	False Negatives
2	5	756	0

## References

[1] Bureau of Labor Statistics (2016). American Time Use Survey. http://www.bls.gov/tus/.

[2] Hu Y., Tilke D., Adams T., Crandall A., Cook D.J., Schmitter-Edgecombe M. (2016). Smart home in a box: Usability study for a large scale self-installation of smart home technologies. J. Reliab. Intell. Environ..

[3] Elfaham A., Hagras H., Helal S., Hossain S., Lee J., Cook D.J. A fuzzy based verification agent for the Persim human activity simulator in ambient intelligent environments. Proceedings of the IEEE International Conference on Fuzzy Systems.

[4] Cook D.J., Das S. (2012). Pervasive computing at scale: Transforming the state of the art. Pervasive Mob. Comput..

[5] Krishnan N., Cook D.J. (2014). Activity recognition on streaming sensor data. Pervasive Mob. Comput..

[6] Cook D.J., Krishnan N., Rashidi P. (2013). Activity discovery and activity recognition: A new partnership. IEEE Trans. Syst. Man Cybern. B.

[7] Cook D.J. (2012). Learning setting-generalized activity models for smart spaces. IEEE Intell. Syst..

[8] Crandall A., Cook D.J. (2013). Behaviometrics for multiple residents in a smart environment. Human Aspects in Ambient Intelligence.

[9] Aggarwal J.K., Ryoo M.S. (2011). Human activity analysis: A review. ACM Comput. Surv..

[10] Chen L., Hoey J., Nugent C.D., Cook D.J., Yu Z. (2012). Sensor-based activity recognition. IEEE Trans. Syst. Man Cybern. C Appl. Rev..

[11] Ke S.-R., Thuc H.L.U., Lee Y.-J., Hwang J.-N., Yoo J.H., Choi K.-H. (2013). A review on video-based human activity recognition. Computers.

[12] Bulling A., Blanke U., Schiele B. (2014). A tutorial on human activity recognition using body-worn inertial sensors. ACM Comput. Surv..

[13] Reiss A., Stricker D., Hendeby G. Towards robust activity recognition for everyday life: Methods and evaluation. Proceedings of the 2013 7th International Conference on Pervasive Computing Technologies for Healthcare.

[14] Vishwakarma S., Agrawal A. (2013). A survey on activity recognition and behavior understanding in video surveillance. Vis. Comput..

[15] Lara O., Labrador M.A. (2013). A survey on human activity recognition using wearable sensors. IEEE Commun. Surv. Tutor..

[16] Chen L., Khalil I., Chen L., Nugent C.D., Biswas J., Hoey J. (2011). Activity recognition: Approaches, practices and trends. Activity Recognition in Pervasive Intelligent Environments.

[17] Tuaraga P., Chellappa R., Subrahmanian V.S., Udrea O., Turaga P. (2008). Machine recognition of human activities: A survey. IEEE Trans. Circuits Syst. Video Technol..

[18] Alon J., Athitsos V., Yuan Q., Sclaroff S. (2008). A unified framework for gesture recognition and spatiotemporal gesture segmentation. IEEE Trans. Pattern Anal. Mach. Intell..

[19] Iglesias J.A., Angelov P., Ledezma A., Sanchis A. (2010). Human activity recognition based on evolving fuzzy systems. Int. J. Neural Syst..

[20] Liao I.L., Fox D., Kautz H. Location-based activity recognition using relational Markov networks. Proceedings of the International Joint Conference on Artificial Intelligence.

[21] Guenterberg E., Ghasemzadeh H., Jafari R. (2012). Automatic segmentation and recognition in body sensor networks using a hidden Markov model. ACM Trans. Embed. Comput. Syst..

[22] Doppa J.R., Fern A., Tadepalli P. (2014). Structured prediction via output space search. J. Mach. Learn. Res..

[23] Doppa J.R., Fern A., Tadepalli P. (2014). HC-Search: Learning heuristics and cost functions for structured prediction. J. Artif. Intell. Res..

[24] Hagras H., Doctor F., Lopez A., Callaghan V. (2007). An incremental adaptive life long learning approach for type-2 fuzzy embedded agents in ambient intelligent environments. IEEE Trans. Fuzzy Syst..

[25] Munguia-Tapia E., Intille S.S., Larson K. Activity recognition in the home using simple and ubiquitous sensors. Proceedings of the International Conference on Pervasive Computing.

[26] Jarafi R., Sastry S., Bajcsy R. (2009). Distributed recognition of human actions using wearable motion sensor networks. J. Ambient Intell. Smart Environ..

[27] Junker H., Amft O., Lukowicz P., Groster G. (2008). Gesture spotting with body-worn inertial sensors to detect user activities. Pattern Recognit..

[28] Maurer U., Smailagic A., Siewiorek D., Deisher M. Activity recognition and monitoring using multiple sensors on different body positions. Proceedings of the International Workshop on Wearable and Implantable Body Sensor Networks.

[29] Gu T., Chen S., Tao X., Lu J. (2010). An unsupervised approach to activity recognition and segmentation based on object-use fingerprints. Data Knowl. Eng..

[30] Philipose M., Fishkin K.P., Perkowitz M., Patterson D.J., Hahnel D., Fox D., Kautz H. (2004). Inferring activities from interactions with objects. IEEE Pervasive Comput..

[31] Gyorbiro N., Fabian A., Homanyi G. (2008). An activity recognition system for mobile phones. Mob. Netw. Appl..

[32] Kwapisz J., Weiss G., Moore S. Activity recognition using cell phone accelerometers. Proceedings of the International Workshop on Knowledge Discovery from Sensor Data.

[33] Candamo J., Shreve M., Goldgof D., Sapper D., Kasturi R. (2010). Understanding transit scenes: A survey on human behavior recognition algorithms. IEEE Trans. Intell. Transp. Syst..

[34] Forster K., Monteleone S., Calatroni A., Roggen D., Troster G. Incremental kNN classifier exploiting correct-error teacher for activity recognition. Proceedings of the International Conference on Machine Learning and Applications.

[35] Bao L., Intille S. Activity recognition from user annotated acceleration data. Proceedings of the International Conference on Pervasive Computing.

[36] Ravi N., Dandekar N., Mysore P., Littman M.L. Activity recognition from accelerometer data. Proceedings of the 17th conference on Innovative Applications of Artificial Intelligence.

[37] Ward J.A., Lukowicz P., Troster G., Starner T.E. (2006). Activity recognition of assembly tasks using body-worn microphones and accelerometers. IEEE Trans. Pattern Anal. Mach. Intell..

[38] Singla G., Cook D.J., Schmitter-Edgecombe M. (2010). Recognizing independent and joint activities among multiple residents in smart environments. Ambient Intell. Humaniz. Comput. J..

[39] Lester J., Choudhury T., Kern N., Borriello G., Hannaford B. A hybrid discriminative/generative approach for modeling human activities. Proceedings of the International Joint Conference on Artificial Intelligence.

[40] Amft O., Troster G. (2009). On-body sensing solutions for automatic dietary monitoring. IEEE Pervasive Comput..

[41] Zhang M., Sawchuk A.A. Motion primitive-based human activity recognition using a bag-of-features approach. Proceedings of the ACM SIGHIT International Health Informatics Symposium.

[42] Blanke U., Schiele B., Kreil M., Lukowicz P., Sick B., Gruber T. All for one or one for all? Combining heterogeneous features for activity spotting. Proceedings of the IEEE International Conference on Pervasive Computing and Communications Workshops.

[43] Van Kasteren T., Noulas A., Englebienne G., Krose B. Accurate activity recognition in a home setting. Proceedings of the ACM Conference on Ubiquitous Computing.

[44] Bulling A., Ward J.A., Gellersen H. (2012). Multimodal recognition of reading activity in transit using body-worn sensors. ACM Trans. Appl. Percept..

[45] Wang S., Pentney W., Popescu A.M., Choudhury T., Philipose M. Common sense based joint training of human activity recognizers. Proceedings of the International Joint Conference on Artificial Intelligence.

[46] Lester J., Choudhury T., Borriello G. A practical approach to recognizing physical activities. Proceedings of the International Conference on Pervasive Computing.

[47] Niu F., Abdel-Mottaleb M. HMM-based segmentation and recognition of human activities from video sequences. Proceedings of the IEEE International Conference on Multimedia and ExpoICME.

[48] Duchenne O., Laptev I., Sivic J., Bach F., Ponce J. Automatic annotation of human activities in video. Proceedings of the International Conference on Computer Vision.

[49] Zheng Y., Wong W.-K., Guan X., Trost S. Physical activity recognition from accelerometer data using a multi-scale ensemble method. Proceedings of the Innovative Applications of Artificial Intelligence Conference.

[50] Hong X., Nugent C.D. (2013). Segmenting sensor data for activity monitoring in smart environments. Pers. Ubiquitous Comput..

[51] Palmes P., Pung H.K., Gu T., Xue W., Chen S. (2010). Object relevance weight pattern mining for activity recognition and segmentation. Pervasive Mob. Comput..

[52] Yamasaki T., Aizawa K. (2007). Motion segmentation and retrieval for 3D video based on modified shape distribution. EURASIP J. Appl. Signal Process..

[53] Keogh E., Chu S., Hart D., Pazzani M. An online algorithm for segmenting time series. Proceedings of the IEEE International Conference on Data Mining.

[54] Crandall A., Cook D.J. (2009). Coping with multiple residents in a smart environment. J. Ambient Int. Smart Environ..

[55] Seymore K., McCallum A., Rosenfeld R. Learning Hidden Markov Model Structure for Information Extraction. Proceedings of the AAAI Workshop on Machine Learning for Information Extraction.

[56] Christiansen H., Dahmcke C.M. A machine learning approach to test data generation: A case study in evaluation of gene finders. Proceedings of the Machine Learning and Data Mining in Pattern Recognition.

[57] Wang H.-C. Modeling idea generation sequences using hidden Markov models. Proceedings of the Annual Meeting of the Cognitive Science Society.

[58] Degottex G., Lanchantin P., Gales M.J.F. A pulse model in log-domain for a uniform synthesizer. Proceedings of the Speech Synthesis Workshop.

[59] Thomas B., Crandall A. A demonstration of PyViz, a flexible smart home visualization tool. Proceedings of the IEEE International Conference on Pervasive Computing and Communication.

[60] Chandola V., Banerjee A., Kumar V. (2009). Anomaly detection: A survey. ACM Comput. Surv..

[61] Ali H., Amalarethinam D.G. (2014). Detecting abnormality in activites performed by people with dementia in smart environment. Int. J. Comput. Sci. Inf. Technol..

[62] Dawadi P., Cook D.J., Schmitter-Edgecombe M. (2016). Automated clinical assessment from smart home-based behavior data. IEEE J. Biomed. Health Inform..

[63] Mahmoud S., Lotfi A., Langensiepen C. Abnormal Behaviours Identification for An Elder’s Life Activities using Dissimilarity Measurements Location based Anomaly Focus on Different Distance Measures. Proceedings of the International Conference on Pervasive Technologies Related to Assistive Environments.

[64] Dodge H.H., Mattek N.C., Austin D., Hayes T.L., Kaye J.A. (2012). In-home walking speeds and variability trajectories associated with mild cognitive impairment. Neurology.

[65] Das B., Cook D.J., Krishnan N., Schmitter-Edgecombe M. (2016). One-class classification-based real-time activity error detection in smart homes. IEEE J. Sel. Top. Signal Process..

[66] Liu F.T., Ting K.M., Zhou Z.-H. Isolation forest. Proceedings of the IEEE International Conference on Data Mining.

[67] Guha S., Mishra N., Roy G., Schrijvers O. Robust random cut forest based anomaly detection on streams. Proceedings of the International Conference on Machine Learning.

[68] Chitnis S., Deshpande N., Shaligram A. (2016). An investigative study for smart home security: Issues, challenges and countermeasures. Wirel. Sens. Netw..

[69] Ring (2016). Never Miss a Visitor. With Ring, You’re always Home. https://ring.com/.

[70] Icontrol Networks (2016). Home Security. https://getpiper.com/howitworks/.

[71] Zhuang X., Huang J., Potamianos G., Hasegawa-Johnson M. Acoustic fall detection using Gaussian mixture models and GMM supervectors. Proceedings of the IEEE International Conference on Acoustics, Speech, and Signal Processing.

[72] Moncrieff S., Venkatesh S., West G., Greenhill S. (2007). Multi-modal emotive computing in a smart house environment. Pervasive Mob. Comput..

[73] Jain A.K., Nandakumar K. (2012). Biometric authentication: System security and user privacy. IEEE Comput..

[74] Euronews (2016). Smarter Home Security Camera Recognises Intrduers Says Maker. http://www.euronews.com/2016/08/03/smarter-home-security-camera-recognises-intruders-says-maker.

[75] Andersson V., Dutra R., Araujo R. Anthropometric and human gait identification using skeleton data from Kinect sensor. Proceedings of the ACM Symposium on Applied Computing.

[76] Helal A., Mann W., Elzabadani H., King J., Kaddourah Y., Jansen E. (2005). Gator Tech Smart House: A Programmable pervasive space. IEEE Comput. Mag..

[77] Jenkins J., Ellis C. Using ground reaction forces from gait analysis: Body mass as a weak biometric. Proceedings of the International Conference on Pervasive Computing.

[78] Watanabe K., Kurihara Y., Tanaka H. (2009). Ubiquitous health monitoring at home-sensing of human biosignals on flooring, on tatami mat, in the bathtub, and in the lavatory. IEEE Sens. J..

[79] Cuddihy P., Weisenberg J., Graichen C., Ganesh M. Algorithm to automatically detect abnormally long periods of inactivity in a home. Proceedings of the ACM SIGMOBILE International Workshop on Systems and Networking Support for Healthcare and Assisted Living Environments.

[80] Aran O., Sanchez-Cortes D., Do M.T., Gatica-Perez D. Anomaly detection in elderly daily behavior in ambient sensing environments. Proceedings of the International Workshop on Human Behavior Understanding Human Behavior Understanding.

[81] Virone G. (2009). Assesing everday life behavioral rythms for the older generation. Pervasive Mob. Comput..

[82] Novak M., Jakab F., Lain L. (2013). Anomaly detection in user daily patterns in smart-home environment. J. Sel. Areas Health Inform..

[83] Ordonez F., de Toldeo P., Sanchis A. (2015). Sensor-based Bayesian detection of anomalous living patterns in a home setting. Pers. Ubiquitous Comput..

[84] Teoh C., Tan C. A neural network approach towards reinforcing smart home security. Proceedings of the Asia-Pacific Symposium on Information and Telecommunication Technologies.

[85] Aicha A.N., Englebienne G., Krose B. Modeling visit behaviour in smart homes using unsupervised learning. Proceedings of the ACM Conference on Ubiquitous Computing.

[86] Han Y., Han M., Lee S., Sarkar A.M.J., Lee Y.-K. (2012). A framework for supervising lifestyle diseases using long-term activity monitoring. Sensors.

[87] Williams J., Cook D. (2017). Forecasting behavior in smart homes based on past sleep and wake patterns. Technol. Health Care.

[88] Mocanu I., Florea A.M. A model for activity recognition and emergency detection in smart environments. Proceedings of the International Conference on Ambient Computing, Applications, Services and Technologies.

[89] Cardinaux F., Brownsell S., Hawley M., Bradley D. (2008). Modelling of behavioural patterns for abnormality detection in the context of lifestyle reassurance. Prog. Pattern Recognit. Image Anal. Appl..

[90] Elbert D., Storf H., Eisenbarth M., Unalan O., Schmitt M. An approach for detecting deviations in daily routine for long-term behavior analysis. Proceedings of the 2011 5th International Conference on Pervasive Computing Technologies for Healthcare (Pervasive Health).

[91] Mori T., Fujii A., Shimosaka M., Noguchi H., Sato T. Typical behavior patterns extraction and anomaly detection algorithm based on accumulated home sensor data. Proceedings of the Conference on Future Generation Communication and Networking.

